# The effect of surface treatments on the bond strength of polyetheretherketone posts: a systematic review protocol

**DOI:** 10.12688/f1000research.154750.1

**Published:** 2024-08-22

**Authors:** Hanen Boukhris, Aymen Ben Hadj Khalifa, Hayet Hajjami, Souha Boudegga Ben Youssef

**Affiliations:** 1 Department of prosthodontics, LR12SP10, University of Monastir, Faculty of Dental Medicine, Monastir, Monastir, Tunisia; 2 Department of Dental Anatomy, University of Monastir, Faculty of Dental Medicine, Monastir, Monastir, Tunisia; 3 department of oral surgery, LR12SP10, University of Monastir, Faculty of Dental Medicine, Monastir, Monastir, Tunisia

**Keywords:** Polyetheretherketone (PEEK), Posts, Surface treatments, Bond strength, Systematic review

## Abstract

**Background:**

Polyetheretherketone (PEEK) is widely used in the biomedical field due to its outstanding biological and mechanical properties. Originally employed as a temporary abutment in implantology, recent research has expanded its indications for more definitive applications, such as frameworks and dental post and core. This shift requires a thorough assessment of PEEK’s adhesion and mechanical characteristics. However, PEEK’s inert properties and intricate chemistry create difficulties in surface treatment, resulting in reduced surface energy and inadequate adhesion. Inducing specific physical and chemical changes aims to overcome these challenges and enhance adhesion for PEEK. Despite its numerous clinical trials, standardized protocols remain lacking. This systematic review aims to assess the impact of surface treatments on the bonding performance of PEEK posts.

**Methods:**

A detailed search of the literature will be conducted across several databases including PubMed, Scopus and clinical trial registries. Additional databases such as Cochrane Central, EMBASE, Web of Science and EBSCO will also be included. The search strategy will target controlled randomized studies and non-randomized clinical trials evaluating the impact of surface treatments on PEEK post adhesion strength. The Newcastle-Ottawa Scale (NOS) will be used to assess bias in non-randomized studies, while the Cochrane Risk of Bias (ROB II) tool will be employed for evaluating randomized controlled trials. Data extraction will focus on study design, treatment methods, outcomes and results.

This systematic review protocol will adhere to the guidelines for systematic reviews outlined in the Preferred Reporting Items for Systematic Reviews and Meta-Analyses (PRISMA).

**Discussion:**

The discussion will explore the implications of findings on clinical practice, highlighting the importance of enhancing PEEK’s bioactivity and surface energy to improve bonding efficacy in dental procedures. Moreover, it will suggest areas for future research to advance dental materials science, aiming to optimize the utilization of PEEK in dental applications

**Systematic review registration:**

PROSPERO: CRD42024529783 (Registered on 08/04/2024).

## Introduction

Endodontically treated teeth (ETT) with over 50% loss of coronal structure are prone to shear forces during chewing and often necessitate post-and-core placement. Posts are employed to secure the core material, enhancing the stability and retention of the final restoration. ^1^^,^^2^

The growing demand for aesthetic improvements in dental treatments has led to the widespread use of prefabricated fiber posts. These posts offer advantages like uniform stress distribution, biocompatibility, and ease of handling, making them preferable over metal posts for restoring endodontically treated teeth. However, they can cause mechanical stress at the restoration margin and fail to strengthen the tooth structure.

Despite their lower elasticity modulus compared to metal posts, fiber posts still exhibit significantly greater stiffness than dentin. Fiber post and core buildup materials can fail due to several mechanisms, such as cracking of the resin matrix, fracture of the fibers, and detachment at the interface.

A novel material with both low Young’s modulus and satisfactory aesthetics has emerged. Thermoplastic polymer Polyetheretherketone (PEEK) has excellent properties. It has a Young’s modulus (3-4 GPa) lower than that of dentin which helps reduce stress on both restorations and teeth. These qualities make PEEK suitable for various dental applications including post and core systems. ^3^^–^^6^

However, the achievement of adhesion between PEEK and resin materials is challenging due to its low surface energy and resistant surface modification. Various treatments including chemical and micromechanical methods are recommended to improve the bonding of composite resin to PEEK posts. ^7^^–^^14^

The goal of this systematic review is to assess how effectively PEEK posts perform with various surface treatments. This is crucial for dental professionals as it provides valuable information for developing dependable bonding protocols for PEEK posts and cores.

## Protocol

### Methods

This protocol outlines the process for conducting a systematic review in accordance with the Preferred Reporting Items for Systematic Reviews and Meta-Analyses (PRISMA) guidelines. ^15^^,^^16^ The methodology from the F1000 journal will be followed to ensure accuracy and consistency at every step. The review protocol is registered in PROSPERO (CRD42024531175) since April 8, 2024.

### Objectives

**Primary objective:** To evaluate the effectiveness of various surface treatments in the improvement of bonding strength for Polyetheretherketone (PEEK) posts.

**Secondary objectives**:

- To provide evidence-based recommendations for clinicians in the choice of surface treatments for PEEK posts during bonding procedures.
- To identify gaps in the literature and to propose possible suggestions for further research.

### Inclusion criteria

The inclusion criteria are structured according to the PICOS model. This model is designed to specify the key components of the research question.

The research question for this systematic review is: What is the effect of various surface treatments on retention and bond strength of polyetheretherketone (PEEK) posts used in dental restoration compared to untreated PEEK posts?

• **Types of participants**: This systematic review will focus on patients requiring dental restoration with polyetheretherketone (PEEK) posts. Eligible participants must be free from medical conditions affecting bone healing, avoid parafunctional habits such as bruxism and have no occlusal problems.
• **Intervention types:** This systematic review will explore PEEK (Polyetheretherketone) use in dental posts. Patients will receive interventions involving surface treatments like etching with 98% sulfuric acid and sandblasting with 50 μm alumina oxide (Al2O3).
• **Types of outcomes:** This systematic review will focus on evaluating PEEK posts in dental restorations with different surface treatments before bonding. Key outcomes include retention rate, fracture resistance compared to traditional posts, and marginal adaptation quality. It will also assess bond strength between PEEK posts and dental materials, post-operative sensitivity, restoration longevity and clinical success rate overall.
• **Measures of effect:** This systematic review will measure effects using quantitative and qualitative assessments. Quantitative measures include statistical analysis of retention rates, fracture resistance, bond strength and restoration longevity.
  Qualitative measures involve evaluating marginal adaptation quality, post-operative sensitivity and clinical success rates through observational data and patient-reported outcomes. Meta-analytical techniques may be used to synthesize findings across studies for comparison.
• **Study types:** Included articles will mainly include randomized controlled trials and prospective or retrospective cohort studies. These studies specifically investigate the use of polyetheretherketone (PEEK) posts in dental restorations, focusing on surface treatments. They are chosen for their substantial data and rigorous methodology to meet the research objectives effectively.
  Excluded articles will cover case reports, case series, abstracts, discussions, interviews, editorials, and opinion pieces, along with research that does not center on PEEK posts or surface treatments. Additionally, studies lacking adequate data or methodology will be omitted to ensure the review’s reliability and relevance.

### Search strategy

A combination of keywords and precise subject headings relevant to the topic will be employed in the refined search strategy, ensuring a comprehensive exploration of pertinent literature. In addition to MEDLINE, databases such as Web of Science, EBSCO, Scopus, Cochrane Central and EMBASE will be meticulously searched to reduce the likelihood of missing relevant studies.

To achieve comprehensiveness, attempts will be made to locate grey literature and active clinical trials through sources such as dissertations, conference proceedings and clinical trial registries. The expert panel will offer guidance in identifying grey literature sources and evaluating their pertinence to the review. ^17^

Furthermore, reference lists of included studies will be systematically examined as part of the search strategy to identify supplementary articles not retrieved solely through electronic databases.

This approach aims to reduce publication bias and ensure a thorough review of the available evidence.

### Study selection

For this systematic review, the study selection process will involve a comprehensive search across all identified databases. Two independent reviewers will screen titles and abstracts to exclude irrelevant studies. Full-text articles of potentially eligible studies will be assessed using predefined inclusion criteria. These criteria will focus on studies that evaluate surface treatments of PEEK in dental post applications.

Reviewers will check article reference lists for additional relevant studies. Articles meeting inclusion criteria will proceed to data extraction.

During screening, any discrepancies among reviewers will be resolved through discussion. If needed, an additional reviewer (HH) will be consulted to ensure accuracy and consensus.

### Evaluation of methodological quality and risk of bias

To guarantee the reliability of the findings, the methodological quality and risk of bias of the included studies will be evaluated using standardized tools. ^18^

For randomized controlled trials, the Cochrane Risk of Bias (ROB II) tool will be utilized to assess methodological rigor across key domains: randomization procedures, adherence to intended interventions, completeness of outcome data, outcome measurement and accuracy in reporting results. Each domain will be categorized as having low, high or unclear risk of bias. ^19^

The quality assessment of non-randomized studies will be conducted using the Newcastle-Ottawa Scale (NOS) which evaluates them based on three main criteria: the selection of study participants, group comparability and outcome ascertainment. Each study will be scored on these criteria with higher scores indicating better methodological quality. ^20^

Two independent reviewers will conduct the assessments to minimize bias and enhance reliability. Any discrepancies in the assessments will be resolved through discussion or by consulting an additional reviewer (HH) to reach a consensus.

Methodological rigor and assessment of bias will ensure that the review’s conclusions are based on high-quality evidence.

### Extraction of data

**Data items:** The following details will be extracted from the selected studies: participant demographics, specifics of the interventions, outcome measures, study characteristics (publication year, author, study design …) and results pertaining to bond strength and surface characteristics.

**Extraction method:** A standardized data extraction form will be created in a Microsoft Excel sheet to systematically capture relevant data from each included study.

Data extraction will be conducted by two reviewers working autonomously to ensure consistent and precise handling of the information.

Discussion will be initiated to resolve any discrepancies, and if needed, input will be sought from a third reviewer (HH).

This structured approach will guarantee thorough and dependable data extraction for subsequent analysis.

### Analysis and synthesis of data

The extracted data will be rigorously analyzed and synthesized to assess the effectiveness of surface treatments for PEEK in dental post materials. Initial descriptive analysis will summarize study details, participant characteristics, intervention particulars and outcome measures such as bond strength and surface characteristics. Quantitative synthesis, including meta-analysis where feasible, will calculate effect sizes with 95% confidence intervals and assess heterogeneity across studies using statistical tests such as Tau-squared, Cochran’s Q test and I-squared, systematically categorized to understand the range of variability.

Subgroup analyses will explore variations in treatment methods and material types. Sensitivity analyses will test result robustness, and qualitative synthesis will offer a narrative summary where quantitative synthesis is not possible. Findings will be interpreted in the context of clinical relevance, discussing methodological strengths and limitations while proposing directions for future research in optimizing PEEK’s performance in dental applications. Forest plot will be used to depict the results, providing a concise visualization of aggregated study effects. ^21^

## Discussion

The outcomes of this systematic review will be highly relevant for practitioners focused on aesthetic and digital dentistry, especially in the management of damaged teeth. By evaluating the efficacy of surface treatments for polyetheretherketone (PEEK) in dental post materials, this review aims to provide evidence-based guidance on enhancing bond strength and surface characteristics critical for durable dental restorations. The findings are anticipated to inform clinical decision-making, facilitating the selection of optimal surface treatment strategies to improve the longevity and aesthetic outcomes of PEEK-based restorations. Moreover, this review will identify areas where current research is lacking and propose avenues for future investigation, aiming to advance the field of dental materials science and enhance patient care in aesthetic and functional dental rehabilitation.

### Ethics and dissemination

No ethical approval is needed for this systematic survey. The authors intend to present the findings at target conferences and publish the research findings in a peer-reviewed journal adopting open science practices.

### Study status

This systematic review is currently in the data analysis process. The protocol of this systematic review was submitted to PROSPERO registry on 8th April, 2024 (CRD42024529783).

## Data Availability

No data are associated with this article. Figshare: PRISMA-P checklist for The Effect of Surface Treatments on the Bond Strength of Polyetheretherketone Posts: A Systematic Review protocol, https://doi.org/10.6084/m9.figshare.25771182.v1. ^22^ Data are available under the terms of the Creative Commons Attribution 4.0 International license (CC-BY 4.0).
