# The effect of surface treatments on the bond strength of polyetheretherketone posts: a systematic review protocol

**DOI:** 10.12688/f1000research.154750.3

**Published:** 2025-04-03

**Authors:** Hanen Boukhris, Aymen Ben Hadj Khalifa, Hayet Hajjami, Souha Boudegga Ben Youssef

**Affiliations:** 1 Department of prosthodontics, LR12SP10, University of Monastir, Faculty of Dental Medicine, Monastir, Monastir, Tunisia; 2 Department of Dental Anatomy, University of Monastir, Faculty of Dental Medicine, Monastir, Monastir, Tunisia; 3 department of oral surgery, LR12SP10, University of Monastir, Faculty of Dental Medicine, Monastir, Monastir, Tunisia

**Keywords:** Polyetheretherketone (PEEK), Posts, Surface treatments, Bond strength, Systematic review

## Abstract

**Background:**

Polyetheretherketone (PEEK) is widely used in the biomedical field due to its outstanding biological and mechanical properties. Originally employed as a temporary abutment in implantology, recent research has expanded its indications for more definitive applications, such as frameworks and dental post and core. However, PEEK’s inert nature and low surface energy pose challenges for adhesion, necessitating surface modifications. Various physical and chemical modification techniques, including acid etching (e.g., 98% sulfuric acid), sandblasting with alumina oxide (Al₂O₃), plasma treatment, laser irradiation, silanization, and air abrasion with silica-coated particles, have been proposed to enhance PEEK’s bonding performance. Despite numerous clinical investigations, standardized protocols for surface treatment remain lacking. This systematic review aims to assess the impact of surface treatments on the bonding performance of PEEK posts.

**Methods:**

A detailed search of the literature will be conducted across several databases including PubMed, Scopus and clinical trial registries. Additional databases such as Cochrane Central, EMBASE, Web of Science and EBSCO will also be included. The search strategy will target controlled randomized studies and non-randomized clinical trials evaluating the impact of surface treatments on PEEK post adhesion strength. The Newcastle-Ottawa Scale (NOS) will be used to assess bias in non-randomized studies, while the Cochrane Risk of Bias (ROB II) tool will be employed for evaluating randomized controlled trials. Data extraction will focus on study design, treatment methods, outcomes and results.

This systematic review protocol will adhere to the guidelines for systematic reviews outlined in the Preferred Reporting Items for Systematic Reviews and Meta-Analyses (PRISMA).

**Discussion:**

The discussion will explore the implications of findings on clinical practice, highlighting the importance of enhancing PEEK’s bioactivity and surface energy to improve bonding efficacy in dental procedures. Moreover, it will suggest areas for future research to advance dental materials science, aiming to optimize the utilization of PEEK in dental applications

**Systematic review registration:**

PROSPERO: CRD42024529783 (Registered on 08/04/2024).

## Introduction

Polyetheretherketone (PEEK) is a high-performance thermoplastic polymer that has gained attention in dentistry due to its unique properties. Originally introduced in the late 20th century for biomedical applications, PEEK is a semicrystalline polymer composed of aromatic rings linked by ketone and ether functional groups. This chemical structure imparts high thermal stability, excellent biocompatibility, and resistance to chemical degradation. One of its most notable advantages in dentistry is its low Young’s modulus (3-4 GPa), which is closer to that of dentin compared to conventional restorative materials like metals and ceramics. These characteristics make PEEK a viable option for multiple dental applications, including implant abutments, removable prostheses, and post-and-core systems. ^1^^,^^2^

The use of PEEK in post-and-core restorations has gained significant interest due to its mechanical properties and aesthetic benefits. Endodontically treated teeth (ETT) with significant coronal structure loss require reinforcement to withstand functional forces, making post placement a necessity in many cases. Traditionally, metal and fiber posts have been employed to enhance retention and stability. However, metal posts, despite their superior strength, have aesthetic limitations and may induce stress concentration, leading to root fractures. Fiber posts, on the other hand, offer improved aesthetics and better stress distribution but exhibit mechanical weaknesses such as resin matrix cracking, fiber fracture, and interfacial debonding. Given these limitations, PEEK posts, available in both prefabricated and custom-made forms, present an alternative that balances mechanical performance with aesthetic considerations. ^3^^–^^6^

Despite its promising properties, PEEK’s clinical application as a post material is hindered by its inherent hydrophobicity and low surface energy, which reduce adhesion to resin-based materials. Unlike conventional post materials such as metal, zirconia, and fiber-reinforced composite posts, PEEK requires surface treatments to improve bonding. Various chemical and micromechanical surface modification techniques, including plasma treatment, acid etching, sandblasting, and silanization, have been proposed to enhance its adhesive properties. ^7^^–^^10^ However, newer advancements in surface modification, such as nanotechnology-based coatings and bioactive materials, offer promising alternatives for improving bonding performance. These modern treatments aim to enhance the surface properties of PEEK at the nanoscale, improving its interaction with resin cements and core buildup materials, and promoting more durable and long-lasting restorations. ^11^^–^^14^

The goal of this systematic review is to evaluate the efficacy of different surface treatments in optimizing the adhesion of PEEK posts. Understanding the bonding mechanisms of PEEK and its adhesion performance compared to conventional post materials is crucial for dental professionals seeking reliable restorative solutions. By assessing available evidence, this study aims to provide valuable insights into developing clinically effective bonding protocols for PEEK post-and-core restorations.

## Protocol

### Methods

This protocol outlines the process for conducting a systematic review in accordance with the Preferred Reporting Items for Systematic Reviews and Meta-Analyses (PRISMA) guidelines. ^15^^,^^16^ The methodology from the F1000 journal will be followed to ensure accuracy and consistency at every step. The review protocol is registered in PROSPERO (CRD42024529783) since April 8, 2024.

### Objectives

**Primary objective:** To evaluate the effectiveness of various surface treatments in the improvement of bonding strength for Polyetheretherketone (PEEK) posts.

**Secondary objectives:**

- To provide evidence-based recommendations for clinicians in the choice of surface treatments for PEEK posts during bonding procedures.
- To identify gaps in the literature and to propose possible suggestions for further research.

### Inclusion criteria

The inclusion criteria are structured according to the PICOS model. This model is designed to specify the key components of the research question.

The research question for this systematic review is: What is the effect of various surface treatments on retention and bond strength of polyetheretherketone (PEEK) posts used in dental restoration compared to untreated PEEK posts?

• **Types of participants:** This systematic review will focus on patients requiring dental restoration with polyetheretherketone (PEEK) posts. Eligible participants must be free from medical conditions affecting bone healing, avoid parafunctional habits such as bruxism and have no occlusal problems. Both in vitro, in vivo, and clinical studies will be eligible for inclusion to ensure a comprehensive analysis across various settings. Heterogeneity between these study types will be managed by conducting separate subgroup analyses and addressing potential variability in findings.
• **Intervention types:** This systematic review will explore the use of Polyetheretherketone (PEEK) in dental posts, focusing on various surface treatments aimed at improving adhesion to resin-based materials. The interventions will include both chemical and micromechanical modifications. Chemical treatments will involve etching with 98% sulfuric acid, silanization, and plasma treatment to enhance surface reactivity. Micromechanical techniques such as sandblasting with 50 μm alumina oxide (Al _2_O _3_), laser surface modification, and air abrasion with silica-coated particles will also be assessed. In addition to traditional treatments, newer surface treatments like nanotechnology-based coatings and bioactive materials will also be explored. These interventions aim to enhance surface reactivity and promote better bonding between PEEK posts and resin-based cements. The effectiveness of these treatments in promoting bonding between PEEK posts and resin-based cements will be systematically analyzed.
• **Types of outcomes:** This systematic review will focus on evaluating PEEK posts in dental restorations with different surface treatments before bonding. Key outcomes will include:
  • **Bond strength:** Assessed through shear bond strength and push-out bond strength tests to evaluate the adhesive performance between PEEK posts and resin-based materials.
  • **Retention rate:** Measured by analyzing the longevity of bonded posts in clinical and in vitro studies.
  • **Fracture resistance:** Compared to traditional post materials such as metal, fiber, and zirconia, using mechanical stress tests.
  • **Marginal adaptation quality:** Evaluated using scanning electron microscopy (SEM) and microleakage tests to assess the integrity of the restoration interface.
  • **Post-operative sensitivity:** Reported through clinical studies assessing patient comfort after post placement, noting that this will be treated as a secondary or exploratory outcome in in vitro studies.
  • **Restoration longevity and clinical success rate:** Determined by survival analysis and failure modes in long-term studies.
• **Measures of effect:** This systematic review will measure effects using quantitative and qualitative assessments. Quantitative measures include statistical analysis of retention rates, fracture resistance, bond strength and restoration longevity. Standardized outcome reporting (e.g., MPa for bond strength) will be employed to improve comparability across studies.
Qualitative measures involve evaluating marginal adaptation quality, post-operative sensitivity and clinical success rates through observational data and patient-reported outcomes. Meta-analytical techniques may be used to synthesize findings across studies for comparison.
• **Study types:** Included articles will mainly include randomized controlled trials and prospective or retrospective cohort studies. These studies specifically investigate the use of polyetheretherketone (PEEK) posts in dental restorations, focusing on surface treatments. They are chosen for their substantial data and rigorous methodology to meet the research objectives effectively.
Excluded articles will cover case reports, case series, abstracts, discussions, interviews, editorials, and opinion pieces, along with research that does not center on PEEK posts or surface treatments. Additionally, studies lacking adequate data or methodology will be omitted to ensure the review’s reliability and relevance.
• **Non-English studies:** Non-English studies will be included in the review if they meet the predefined inclusion criteria. For non-English studies, the review team will ensure that appropriate translation or interpretation of relevant data is conducted. If no reliable translations are available, such studies may be excluded. This will be discussed in the results section, along with any impact on study conclusions.

### Search strategy

A combination of keywords and precise subject headings relevant to the topic will be employed in the refined search strategy, ensuring a comprehensive exploration of pertinent literature. In addition to MEDLINE, databases such as Web of Science, EBSCO, Scopus, Cochrane Central and EMBASE will be meticulously searched to reduce the likelihood of missing relevant studies.

**Keywords/MeSH terms:**

• PEEK
• Polyetheretherketone
• Surface treatment
• Bonding
• Bond strength
• Adhesion
• Post-and-core
• Dental restoration
• Nanotechnology
• Bioactive materials

A sample search string designed for use across the selected databases is as follows:

**Search string for MEDLINE:**

“(Polyetheretherketone OR PEEK) AND (surface treatment OR etching OR silanization OR plasma treatment OR sandblasting OR laser modification OR nanotechnology OR bioactive materials) AND (bond strength OR adhesion OR bonding) AND (dental restoration OR post and core).”

**Search string for scopus:**

“(PEEK OR Polyetheretherketone) AND (surface treatment OR silanization OR plasma treatment OR acid etching OR sandblasting OR laser treatment) AND (bond strength OR adhesive strength OR bonding) AND (restoration OR post and core OR dental).”

In addition to searching the electronic databases, efforts will be made to locate grey literature and active clinical trials through sources such as dissertations, conference proceedings, and clinical trial registries. An expert panel will offer guidance in identifying grey literature sources and evaluating their pertinence to the review. ^17^

Furthermore, reference lists of included studies will be systematically examined as part of the search strategy to identify supplementary articles not retrieved solely through electronic databases.

This approach aims to reduce publication bias and ensure a thorough review of the available evidence.

### Study selection

For this systematic review, the study selection process will involve a comprehensive search across all identified databases. Two independent reviewers will screen titles and abstracts to exclude irrelevant studies. Full-text articles of potentially eligible studies will be assessed using predefined inclusion criteria. These criteria will focus on studies that evaluate surface treatments of PEEK in dental post applications.

Reviewers will check article reference lists for additional relevant studies. Articles meeting inclusion criteria will proceed to data extraction.

During screening, any discrepancies among reviewers will be resolved through discussion. If needed, an additional reviewer (HH) will be consulted to ensure accuracy and consensus.

### Ethical considerations

As this systematic review aims to synthesize data from existing studies, all included studies must have adhered to ethical standards set by the respective institutional review boards or ethics committees. Specifically, clinical and in vivo studies involving human participants should have received prior ethical approval for patient data collection and use, in accordance with ethical guidelines and regulations such as the Declaration of Helsinki. Data from in vitro studies will be considered as non-human subject research, and ethical compliance will be confirmed by reviewing the ethical approvals provided in the individual studies. No new patient data will be collected as part of this systematic review, and patient confidentiality and informed consent must have been respected in all the included studies. This ensures that the rights and well-being of patients are safeguarded while also maintaining the integrity and ethical standards of the research process.

### Evaluation of methodological quality and risk of bias

To ensure the reliability of the findings, the methodological quality and risk of bias of the included studies will be evaluated using standardized tools.

For randomized controlled trials (RCTs), the Cochrane Risk of Bias (ROB II) tool will be used to assess key methodological domains, including randomization procedures, adherence to intended interventions, completeness of outcome data, outcome measurement, and accuracy in reporting results. Each domain will be classified as having a low, high, or unclear risk of bias. Studies categorized as having an unclear or high risk of bias will be further analyzed through sensitivity analysis to determine their impact on the overall findings. ^18^^,^^19^

For non-randomized studies, the Newcastle-Ottawa Scale (NOS) will be employed to assess quality based on three main criteria: selection of study participants, group comparability, and outcome ascertainment. Each study will be scored based on these criteria, with higher scores indicating better methodological quality. ^20^

Two independent reviewers will conduct the assessments to minimize bias and enhance reliability. Any discrepancies in the evaluations will be resolved through discussion or by consulting an additional reviewer (HH) to reach a consensus. These measures will ensure that the conclusions of the systematic review are based on high-quality evidence.

**Meta-Analysis and handling of heterogeneity:** If the included studies present comparable methodologies and outcome measures, a meta-analysis will be conducted. Heterogeneity among studies will be evaluated using the I ^2^ statistic and Cochran’s Q test. If substantial heterogeneity (I ^2^ > 50%) is detected, subgroup analyses will be performed based on factors such as surface treatment type, study design (in vitro vs. in vivo), and testing methodology. Random-effects models will be applied if heterogeneity is high, while fixed-effects models will be used for homogeneous data. If meta-analysis is not feasible, a qualitative synthesis will be provided to summarize the findings. These approaches will ensure a comprehensive and statistically sound evaluation of the effectiveness of surface treatments for PEEK posts. ^20^

### Extraction of data

**Data items:** The following details will be extracted from the selected studies: participant demographics, specifics of the interventions, outcome measures, study characteristics (publication year, author, study design …) and results pertaining to bond strength and surface characteristics.

**Extraction method:** A standardized data extraction form will be created in a Microsoft Excel sheet to systematically capture relevant data from each included study.

Data extraction will be conducted by two reviewers working autonomously to ensure consistent and precise handling of the information.

Discussion will be initiated to resolve any discrepancies, and if needed, input will be sought from a third reviewer (HH).

This structured approach will guarantee thorough and dependable data extraction for subsequent analysis.

### Analysis and synthesis of data

The extracted data will be rigorously analyzed and synthesized to assess the effectiveness of surface treatments for PEEK in dental post materials. Initial descriptive analysis will summarize study details, participant characteristics, intervention particulars and outcome measures such as bond strength and surface characteristics. Quantitative synthesis, including meta-analysis where feasible, will calculate effect sizes with 95% confidence intervals and assess heterogeneity across studies using statistical tests such as Tau-squared, Cochran’s Q test and I-squared, systematically categorized to understand the range of variability.

Subgroup analyses will explore variations in treatment methods and material types. Sensitivity analyses will test result robustness, and qualitative synthesis will offer a narrative summary where quantitative synthesis is not possible. In vitro studies, typically conducted in controlled laboratory settings, will be evaluated for their relevance to real-world clinical conditions, as they may not fully replicate the complexities of the oral environment (e.g., dynamic forces, and biological factors). In vivo and clinical studies, which reflect real-world conditions, will be appraised for their clinical applicability, with consideration given to patient-specific factors such as age, oral hygiene, and systemic conditions, which could influence bonding outcomes. Where feasible, data from in vitro and in vivo studies will be combined through meta-analytical techniques, adjusting for differences in experimental conditions to provide a unified conclusion. The strengths and limitations of both in vitro and in vivo studies will be discussed in the results section, with an emphasis on how study design impacts the interpretation of bond strength and retention outcomes.

A forest plot will be used to visually represent the aggregated study effects, providing a clear depiction of the results. Finally, the findings will be interpreted in the context of their clinical relevance, with the strengths and weaknesses of the included studies discussed, along with recommendations for clinical practice and directions for future research aimed at optimizing PEEK’s performance in dental applications. ^21^

## Discussion

The outcomes of this systematic review will be highly relevant for practitioners focused on aesthetic and digital dentistry, especially in the management of damaged teeth. By evaluating the efficacy of surface treatments for polyetheretherketone (PEEK) in dental post materials, this review aims to provide evidence-based guidance on enhancing bond strength and surface characteristics critical for durable dental restorations. The findings are anticipated to inform clinical decision-making, facilitating the selection of optimal surface treatment strategies to improve the longevity and aesthetic outcomes of PEEK-based restorations. Moreover, this review will identify areas where current research is lacking and propose avenues for future investigation, aiming to advance the field of dental materials science and enhance patient care in aesthetic and functional dental rehabilitation.

This review differs from or builds upon existing systematic reviews on PEEK bonding in several key ways. First, it aims to provide a more comprehensive evaluation by considering studies from a broad range of sources, including in vitro, in vivo, and clinical studies. Previous reviews may have focused primarily on one type of study design, which limits their ability to assess the generalizability of surface treatment results to real-world clinical settings. Moreover, this review will also examine the specific impact of various surface treatment methods (e.g., etching, plasma treatment, sandblasting) on both bond strength and surface characteristics, providing a more detailed analysis of the effectiveness of these interventions across different experimental conditions. Additionally, by addressing issues such as heterogeneity between study designs and potential confounding factors, this review seeks to offer a more robust synthesis of the evidence than prior systematic reviews on this topic.

### Ethics and dissemination

No ethical approval is needed for this systematic survey. The authors intend to present the findings at target conferences and publish the research findings in a peer-reviewed journal adopting open science practices.

### Study status

This systematic review is currently in the data analysis process. The protocol of this systematic review was submitted to PROSPERO registry on 8th April, 2024 (CRD42024529783).

## Data Availability

No data are associated with this article. Figshare: PRISMA-P checklist for The Effect of Surface Treatments on the Bond Strength of Polyetheretherketone Posts: A Systematic Review protocol, https://doi.org/10.6084/m9.figshare.25771182.v1. ^22^ Data are available under the terms of the Creative Commons Attribution 4.0 International license (CC-BY 4.0).
