# Supplementary material for: The effect of surface treatments on the bond strength of polyetheretherketone posts: a systematic review protocol
Source: F1000Res. 2025 Apr 3;13:951. Originally published 2024 Aug 22. [Version 3] doi: 10.12688/f1000research.154750.3 (PMC12008716; doi:10.12688/f1000research.154750.3)
Supplement: Peer review history

# Reviewer report: Reviewer response for version 3

**DOI:** 10.5256/f1000research.179536.r376277

**Published:** 2025-04-25

**Authors:** Marwa M Wahsh

**Affiliations:** 1 Prosthodontics, Galala University (Ringgold ID: 577705), Suez, Suez Governorate, Egypt; 2 Fixed Prosthodontics, Faculty of dentistry at Ainshams University, Cairo, Egypt

First, I would thank the Editorial Team, F1000Research, for the invitation to peer review this protocol. Next, I would like to express my appreciation for the authors’ efforts in presenting this systematic review plan.

 Overall view:

 This systematic review protocol aims to evaluate the effectiveness of different surface treatments to improve the bond strength of polyetheretherketone (PEEK) posts, which are increasingly used in dental restorations due to their high biocompatibility and mechanical properties. The authors focus on PEEK's inherent challenges—low surface energy and hydrophobicity—and propose a comprehensive analysis of surface treatment techniques, including acid etching, sandblasting, plasma treatment, and newer nanotechnology-based coatings. The protocol adheres to PRISMA guidelines and is registered in PROSPERO (CRD42024529783), ensuring methodological transparency.

 Objectives:

- Clear objectives.

 Methodology:

- The protocol includes comprehensive search through multiple databases (PubMed, Scopus, Cochrane, EMBASE, etc.) in addition to clinical trials.
- The inclusion criteria are clear, the PICOS framework is appropriately applied. However, the exclusion criteria need to be specific.
- The protocol clarifies how inconsistencies between in vitro and clinical data will be reconciled, which will make data interpretation more valid.
- The clinical feasibility for using sulfuric acid as a surface treatment is debated due to toxicity risks.

Clinical significance:

 The review can be a reference for clinicians to select appropriate surface treatments for different settings, e.g. sulfuric acid etching may be limited for non-clinical settings due to safety concerns, while other alternatives can be used more safely.

 Final Recommendation:

 This systematic review protocol addresses an applicable and clinically relevant question in restorative dentistry. Clinicians should expect the finalized review to provide evidence-based decisions, in addition researchers can use its findings to design new studies on under investigated treatments.

 Future research should prioritize clinical trials comparing long-term performance of surface treatments and safer alternatives to corrosive agents like sulfuric acid.

Is the study design appropriate for the research question?

Yes

Is the rationale for, and objectives of, the study clearly described?

Yes

Are sufficient details of the methods provided to allow replication by others?

Yes

Are the datasets clearly presented in a useable and accessible format?

Yes

Reviewer Expertise:

Fixed prosthodontics- adhesion- ceramics- esthetics- color science

I confirm that I have read this submission and believe that I have an appropriate level of expertise to confirm that it is of an acceptable scientific standard.

# Reviewer report: Reviewer response for version 3

**DOI:** 10.5256/f1000research.179536.r375420

**Published:** 2025-04-18

**Authors:** Retno Ardhani

**Affiliations:** 1 Universitas Gadjah Mada, Jalan, Indonesia

I acknowledge the author's effort to revise, thus increasing the clarity of the work. Vital points were well addressed and explained.

Is the study design appropriate for the research question?

Partly

Is the rationale for, and objectives of, the study clearly described?

Yes

Are sufficient details of the methods provided to allow replication by others?

Partly

Are the datasets clearly presented in a useable and accessible format?

Not applicable

Reviewer Expertise:

NA

I confirm that I have read this submission and believe that I have an appropriate level of expertise to confirm that it is of an acceptable scientific standard.

# Reviewer report: Reviewer response for version 2

**DOI:** 10.5256/f1000research.177690.r371773

**Published:** 2025-03-22

**Authors:** Retno Ardhani

**Affiliations:** 1 Universitas Gadjah Mada, Jalan, Indonesia

The study protocol is confirmed to be registered in the PROSPERO database. The aims of the study are clearly stated and supported by a proper study design. However, the lack of clarity to ensure study replicability is found. Please consider including (and confirm):

 1. The applied keywords combination and what the Boolean operator used. 

 2. Does the combination apply to all databases?

 3. Are there any exclusion criteria applied? 

 4. Graphical description to resume overall steps and selection criteria applied. 

 5. Do you also consider the variability of the resin-based cement material composition used in the research since it also influences adhesion between a post and adhesive cement? How do you manage this variation in your study?

 6. Units that are bound to each parameter/outcome to ensure comparability. For example, how is the post-operative sensitivity evaluated? Is there any specific scale employed?

 7. In this study, various surface modifications will be compared. However, modification on each surface treatment has the potential to result in different surfaces; for example, plasma treatment can be performed using various chemicals, or there are time and particle size modifications for sandblasting. How do you manage this variation? 

 Thank you.

Is the study design appropriate for the research question?

Partly

Is the rationale for, and objectives of, the study clearly described?

Yes

Are sufficient details of the methods provided to allow replication by others?

Partly

Are the datasets clearly presented in a useable and accessible format?

Not applicable

Reviewer Expertise:

Biomedical, Regenerative Therapy, Polymer, Surface Treatment

I confirm that I have read this submission and believe that I have an appropriate level of expertise to state that I do not consider it to be of an acceptable scientific standard, for reasons outlined above.

# Reviewer report: Reviewer response for version 2

**DOI:** 10.5256/f1000research.177690.r366864

**Published:** 2025-02-24

**Authors:** Noha Taymour

**Affiliations:** 1 Department of Substitutive Dental Sciences, College of Dentistry, Imam Abdulrahman Bin Faisal University, Dammam, Saudi Arabia

I would like to thank the authors for their meticulous effort in developing this comprehensive protocol. This systematic review is particularly important as it aims to establish evidence on surface treatment techniques, offering insights that could lead to standardized protocols and improved clinical outcomes. The findings have the potential to bridge gaps in current knowledge and guide clinicians in selecting effective bonding strategies for PEEK posts, ultimately enhancing patient care and restoration longevity. Some points need to be addressed to ensure clarity and reliability of results

 1-    The PROSPERO registration number in the abstract (CRD42024529783) conflicts with the protocol section (CRD42024531175). 

 2-    The introduction overlaps with the abstract’s background section. Streamline to avoid duplication.

 3-    Please provide more comprehensive introduction with focus on the latest surface treatment for posts bonding.

 4-    Each statement should be supported by evidence based reference. Please try to merge multiple sentences into one comprehensive paragraph. 

 5-    The inclusion criteria mention "patients requiring dental restoration," yet bond strength is often tested in vitro. Revise the PICOS framework to state whether in vitro, in vivo, and clinical studies are all eligible. If so, justify how heterogeneity between study types will be managed.

 6-    Please Provide a rationale for excluding patients with certain conditions. For example, explain how these factors might confound bonding outcomes.

 7-    The protocol lacks specific keywords/MeSH terms used. Please include a sample search string in the protocol to demonstrate how terms like "PEEK," "surface treatment," and "bond strength" will be combined.

 8-     There is no mention of whether non-English studies will be included or excluded.

 9-    Address how outcomes like "post-operative sensitivity" will be handled if absent in in vitro studies. Specify whether such outcomes are secondary or exploratory.

 10-    While PROSPERO registration is noted, ethical considerations regarding patient data from included studies are not addressed. The authors could include a statement about ensuring ethical compliance in included studies.

 11-     No discussion is provided on how this review differs from or builds upon existing systematic reviews on PEEK bonding.

 12-    I would suggest including newer surface treatments like nanotechnology-based coatings or bioactive materials to ensure relevance to advancing dental practices.

 13-    Standardize Outcome Reporting:  for example (MPa for bond strength) to improve comparability across studies.

 14-    Acknowledge in the study design the potential challenges in data findings from in vitro vs. in vivo studies and propose strategies to mitigate them.

Is the study design appropriate for the research question?

Yes

Is the rationale for, and objectives of, the study clearly described?

Yes

Are sufficient details of the methods provided to allow replication by others?

Yes

Are the datasets clearly presented in a useable and accessible format?

Yes

Reviewer Expertise:

Prosthodontics, Dental Implants, Polymers, Ceramics, Esthetic Dentistry, Digital Dentistry

I confirm that I have read this submission and believe that I have an appropriate level of expertise to confirm that it is of an acceptable scientific standard, however I have significant reservations, as outlined above.

# Response

**Published:** 2025-03-24

**Authors:** Hanen Boukhris

**Affiliations:** university hospital F- Hached, faculty of dental medicine university of monastir tunisia, sousse, tunisia, Tunisia

We sincerely appreciate the reviewers' thorough and insightful evaluation of our work. Their constructive feedback has been invaluable in refining the study and strengthening its overall quality.

 In response, we have carefully addressed each concern, ensuring consistency in PROSPERO registration, refining the introduction to enhance clarity and conciseness, and reinforcing the methodology to improve robustness. We have also provided additional clarifications on inclusion criteria, search strategies, and ethical considerations, thereby enhancing transparency and reliability. Furthermore, we have incorporated the latest advancements in surface treatment techniques, ensuring that the study remains relevant to current scientific and clinical practices. Standardizing outcome metrics, particularly in bond strength measurement, further improves the study's comparability and applicability.

 We believe these enhancements significantly contribute to the rigor and impact of our work. We remain open to any further recommendations and greatly value the reviewers' expertise and insights in advancing the quality of this research.

# Reviewer report: Reviewer response for version 1

**DOI:** 10.5256/f1000research.169814.r316835

**Published:** 2024-09-18

**Authors:** Marwa Emam

**Affiliations:** 1 Ain Shams University,, , Cairo, Egypt

Review report for the submitted manuscript “The effect of surface treatments on the bond strength of polyetheretherketone posts: a systematic review protocol”

 I would like to thank the authors for submitting their protocol for review. The use of PEEK posts in dental applications is a developing subject, so a systematic assessment of surface treatment procedures is extremely important. PEEK's biocompatibility and mechanical strength make it excellent for dental posts, but the inert surface makes bonding challenging. This review could fill a crucial gap in clinical practice.

 Overall, the language is clear, but minor editing of English language might be required in the final submitted review. Author might want to explore options for refining their text to improve its flow and readability. This can make their article more engaging and clear.

1. Title: -  Approved as is.

2. Abstract: Kindly give a hint on the different surface treatment techniques of PEEK for the reader.  

3. Introduction: -I think the introduction is a bit shallow. I would prefer the introduction to begin with PEEK material (history, chemical composition and properties) as it is the main objective of the study. Then going through the different applications quickly and concentrating on the endodontically treated teeth and post indications and PEEK post (ready & custom made) as one of the options available for clinicians. Finally, stating PEEK adhesion properties as a weakness that different surface treatments aim to improve.

 -Kindly connect short paragraphs into paragraphs of 6-7 lines paragraphs.

 -Kindly add references to the second & third paragraph.

 - The authors could elaborate on the therapeutic relevance of PEEK's bond strength as compared to conventional materials like metal, zirconia or fiber posts.

4. Methods: -The protocol is well-structured and follows known principles for systematic reviews (PRISMA). The use of numerous databases (e.g., PubMed, Scopus, Cochrane Central) will ensure a thorough literature review. The intended use of risk-of-bias tools (ROB II and NOS) and data extraction procedures demonstrates a commitment to evidence of excellent quality synthesis.

Intervention types:

 There is no mention of the surface treatments of PEEK aimed for exploration. Only 2 examples were mentioned so kindly give more details regarding treatments to be studied.

Outcomes:

 More precise definitions would be useful in the outcomes section. While bond strength and retention are indicated, it is unclear how they would be tested. Providing additional details on the anticipated outcomes and their clinical significance could enhance the protocol.

Evaluation of methodological quality and risk of bias:

 -The protocol’s description of bias assessment tools (ROB II and NOS) is good, but how will studies with unclear risks of bias be handled in the analysis? Also kindly give more details regarding the “standardized tools” you have mentioned as means of evaluation.

Meta analysis:

 While the authors highlight the possibility of meta-analysis, it would be interesting to describe how they intend to deal with possible heterogeneity among studies (e.g., differences in surface treatments, study designs).

5. Conclusions: Although this protocol presents a well-designed systematic review that is expected to yield useful information about the surface treatments of PEEK posts in dental applications, it would be strengthened by adding the details addressed in the comments above for further enhancement.

Is the study design appropriate for the research question?

Yes

Is the rationale for, and objectives of, the study clearly described?

Yes

Are sufficient details of the methods provided to allow replication by others?

Yes

Are the datasets clearly presented in a useable and accessible format?

Yes

Reviewer Expertise:

Fixed prosthodontics, implant, occlusion, ceramics, dental materials, esthetics, digital dentistry.

I confirm that I have read this submission and believe that I have an appropriate level of expertise to confirm that it is of an acceptable scientific standard, however I have significant reservations, as outlined above.

# Response

**Published:** 2025-01-29

**Authors:** Hanen Boukhris

**Affiliations:** university hospital F- Hached, faculty of dental medicine university of monastir tunisia, sousse, tunisia, Tunisia

We appreciate the reviewers’ valuable feedback, which has helped improve the clarity, depth, and rigor of our manuscript. Below, we provide detailed responses to each comment and indicate the revisions made accordingly.

**1.Introduction**

1. **Comment:** "The introduction should start with PEEK’s material properties, history, and applications before discussing its role in post-and-core restorations."
**Response:** We have reorganized the introduction to first present PEEK’s chemical composition, mechanical properties, and biomedical applications before focusing on its role in post-and-core restorations. The revised introduction now follows a logical progression, improving the contextual framework of the study.
2. **Comment:** "Please provide more details on the different surface treatment techniques of PEEK."
**Response:** We have expanded the discussion on surface treatments, including plasma treatment, acid etching (98% sulfuric acid), sandblasting (50 μm alumina oxide), laser surface modification, silanization, and air abrasion. These modifications and their rationale are now clearly described.
3. **Comment:** "There is limited discussion on the comparative performance of PEEK posts against conventional materials like metal, zirconia, and fiber posts."
**Response:** The revised manuscript now includes a comparative analysis highlighting the strengths and limitations of PEEK versus conventional post materials, emphasizing its clinical relevance.

1. **Comment:** "How will studies with unclear risk of bias be handled in the analysis?"
**Response:** We have specified that studies with an unclear risk of bias will undergo sensitivity analysis, and their influence on the final conclusions will be carefully considered.
2. **Comment:** "Clarify how heterogeneity among studies will be addressed in the meta-analysis."
**Response:** A heterogeneity assessment will be conducted using I² statistics, and subgroup analysis or meta-regression will be applied if significant heterogeneity is detected.
3. **Comment:** "Provide more precise definitions of key outcomes."
**Response:** We have revised the outcomes section to specify assessment methods for bond strength (e.g., push-out tests), fracture resistance, and marginal adaptation quality.

These responses, along with the corresponding revisions, have strengthened our manuscript and improved its clarity. Thank you for your constructive feedback.
